# The human middle ear in motion: 3D visualization and quantification using dynamic synchrotron-based X-ray imaging

**DOI:** 10.1038/s42003-023-05738-6

**Published:** 2024-02-07

**Authors:** Margaux Schmeltz, Aleksandra Ivanovic, Christian M. Schlepütz, Wilhelm Wimmer, Aaron K. Remenschneider, Marco Caversaccio, Marco Stampanoni, Lukas Anschuetz, Anne Bonnin

**Affiliations:** 1 Paul Scherrer Institute, Swiss Light Source, Villigen, Switzerland; 2 Department of Otorhinolaryngology, Head and Neck Surgery, Inselspital, Bern University Hospital, Bern, Switzerland; 3 Hearing Research Laboratory, ARTORG Center for Biomedical Engineering Research, University of Bern, Bern, Switzerland; 4 TUM School of Medicine, Klinikum rechts der Isar, Department of Otorhinolaryngology, Munich, Germany; 5 Department of Otolaryngology, Head and Neck Surgery, Mass. Eye and Ear, Boston Children Hospital, Harvard Medical School, Boston, MA USA; 6 Institute for Biomedical Engineering, University and ETH Zürich, Zurich, Switzerland

**Keywords:** X-ray tomography, Translational research, Data processing

## Abstract

The characterization of the vibrations of the middle ear ossicles during sound transmission is a focal point in clinical research. However, the small size of the structures, their micrometer-scale movement, and the deep-seated position of the middle ear within the temporal bone make these types of measurements extremely challenging. In this work, dynamic synchrotron-based X-ray phase-contrast microtomography is used on acoustically stimulated intact human ears, allowing for the three-dimensional visualization of entire human eardrums and ossicular chains in motion. A post-gating algorithm is used to temporally resolve the fast micromotions at 128 Hz, coupled with a high-throughput pipeline to process the large tomographic datasets. Seven ex-vivo fresh-frozen human temporal bones in healthy conditions are studied, and the rigid body motions of the ossicles are quantitatively delineated. Clinically relevant regions of the ossicular chain are tracked in 3D, and the amplitudes of their displacement are computed for two acoustic stimuli.

## Abstract (web summary)

Synchrotron X-ray imaging of stimulated human middle ears reveals the 3D movement of auditory ossicles: it enables their motion quantification, confirms the lever-like motions of the malleus and incus and reveals a more intricate stapedial behavior.

## Introduction

Deterioration of hearing is a significant problem in all age categories worldwide. It is estimated that by 2050 over 700 million people will have disabling hearing loss^1^. The middle ear is an air-filled space containing three tiny bones known as the ossicles - the malleus, the incus, and the stapes. The location of the human middle ear within the temporal bone, its size, and the amplitude of its micromotions make studying the sound transmission mechanisms extremely challenging.

The anatomy of the middle ear has traditionally been investigated with histology^2,3^. Although highly accurate, this technique requires sectioning of decalcified tissue and, therefore, bears the risk of structural deformation. Studies using X-ray micro-computed tomography (μCT) have overcome this issue and enabled morphological analyses on intact specimens^4–6^. The contrast and resolution of X-ray imaging could be pushed even further using a more advanced imaging technique, synchrotron-based X-ray phase-contrast microtomography. It relies on the high flux and partial coherence of a powerful synchrotron-based X-ray beam. This technique provides information about both the intensity attenuation and the phase shift of the X-ray beam passing through the sample, revealing soft tissue material that would appear uniform in conventional μCT^7,8^. The first investigation of middle ear structures with this technique in unstained, non-decalcified human temporal bones was published by Elfarnawany et al. (2017), who reported a good visualization of bone and soft tissues with a voxel size of around 9 μm^9^. More recently, Anschuetz et al. investigated the micrometric structure of the auditory ossicles at a voxel size of 0.65 μm, the highest resolution reported in the X-ray investigation of the human auditory system so far^10^.

In addition to morphological studies, the biomechanic investigations of the ossicles have been crucial to understanding the sound transmission mechanism in the middle ear. In 1868, Helmholtz performed one of the first experiments by manipulating the manubrium with a needle tip, and described the middle ear functions as a system of levers that rotate around a single axis. This axis is formed on one side by the anterior mallear ligament and the dorsal fibers of the lateral mallear ligament, and on the other side by the posterior incudal ligament^11^. In 1960, however, von Békésy demonstrated that the incudomalleolar (IM) joint could move the incus biaxially, and that the motion of the IM joint would change with frequencies at physiological sound levels: at lower frequencies of the hearing range, rotations would occur about the above-mentioned axis, while higher frequencies could trigger rotations about a second axis, perpendicular to the first through the IM joint^12,13^.

Velocity and displacement magnitude of the ossicles have been traditionally measured by Laser Doppler Vibrometry^14–16^. It can detect movements in the nanometer range based on the Doppler effect and, therefore, can be used on the middle ear stimulated with a broad range of frequencies between 200–10’000 Hz. However, Laser Doppler Vibrometry requires manual placement of reflective beads on the areas of interest, hindering the repeatability and reproducibility of the measurements across specimens with various sizes and shapes, and it usually provides only one-dimensional measurements. Furthermore, Laser Doppler Vibrometry requires direct sight to the ossicles, which generally includes surgical manipulations that may affect the sound transfer function, like a posterior tympanotomy, removing the vertical segment of the facial nerve, and sectioning the stapedial tendon. Chang et al. (2013) proposed a technique called optical coherence tomography vibrography to simultaneously capture the 3D shape and the sound-induced motions of the tympanic membrane and parts of the ossicular chain^17,18^. But only the motion parallel to the optical beam can be stimulated with this technique, and the true vibration amplitude is underestimated.

To overcome these limitations, a non-destructive 3D visualization of the middle ear motions (4D imaging) with high spatial and temporal resolutions appears helpful to study the biomechanics of the middle ear in its natural state. The TOMCAT beamline of the Swiss Light Source (SLS, PSI) has been specialized in time-resolved X-ray imaging, with the development of an ultra-fast camera that allows dynamic processes to be followed in time and three dimensions^19^. Recently, the beamline was able to provide an acquisition speed reaching 1000 tomograms per second while keeping a spatial resolution of a few micrometers^20^. With a system exhibiting a periodic movement, a retrospective gating strategy could be used to increase even further the temporal resolution up to 3 kHz^21,22^.

In this work, synchrotron-based X-ray phase-contrast microtomography was combined with the ultra-fast capabilities of the TOMCAT end-station to directly observe the vibrations of the intact human middle ear, tympanic membrane (TM) and ossicular chain, in response to acoustic stimulation. Micro-movements of the three ossicles, treated as rigid bodies, were resolved at 128 Hz and quantitatively delineated. Several clinically relevant anatomical points of the ossicular chain were tracked in 3D, and the amplitudes of their motions were computed for various acoustic stimuli. The findings corroborated the lever-like motions of the mallei and incudes, while revealing a more intricate stapedial behavior.

## Results

### Direct motion visualization at a high sound pressure level

A pilot study was conducted on a first fresh-frozen human specimen (*Fresh1*), acoustically stimulated with a pure tone frequency of 127 Hz and with a sound pressure level of 140 dB SPL. These acoustic parameters were chosen in order to establish the first sound stimulation protocol in conditions leading to large ossicle movements, with a high sound pressure level and a frequency low in the hearing range. The dynamic microtomography of the moving sample was performed assuming a periodic vibration of the middle ear with the same period as the sound stimulation. This period was sampled into 10 time steps, each of those called a phase of movement *p*_*j*_. The tomography datasets were built up by acquiring a substantial number of projections over multiple and consecutive motion cycles, while the sample stage slowly rotated, and retrospectively sorting these projections back into the 10 different phases of movement (post-gating process, see Materials and Methods). After reconstruction, 3D static volumes were obtained for each phase of movement, providing a high-resolution visualization of the whole ossicular chain. In addition, phase retrieval methods applied to one dataset allowed to reveal soft tissue between the ossicles (see the incudomalleolar and incudostapedial joints in Supplementary Fig. 1.

**Fig. 1:**
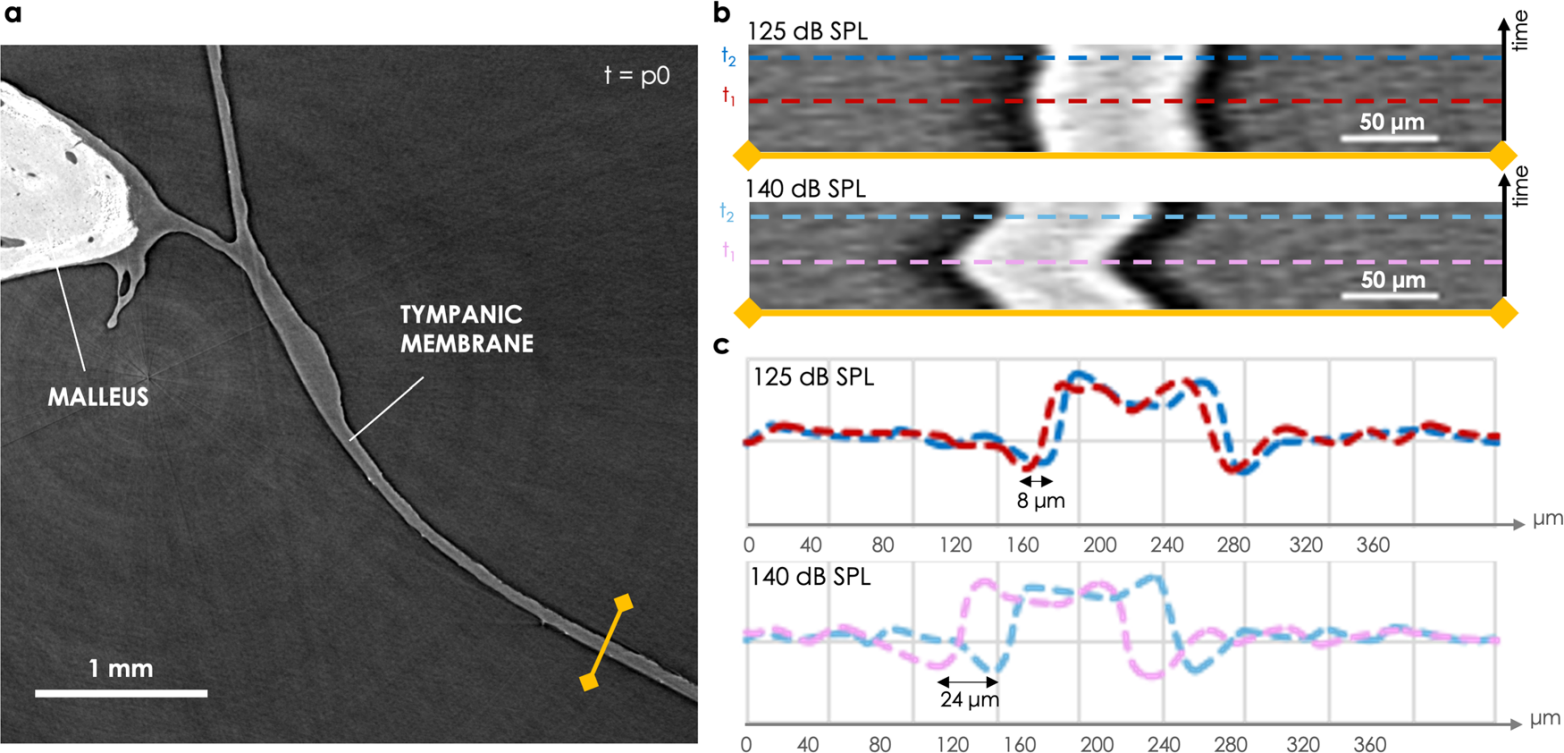
2D visualization of the tympanic membrane (TM) in a fresh-frozen human specimen (*Fresh1*) stimulated at 127 Hz and 2 different sound pressure levels (SPLs). **a** A 2D slice is taken from the 3D volume reconstructed at phase *p*_0_ of the movement. **b** The intensity profile along the yellow line is displayed over time (over the 10 phases of movement), for the 2 stimulation levels, 125 dB SPL and 140 dB SPL. **c** The intensity profiles along the dashed lines in **b** (at two specific times *t*_1_ and *t*_2_ corresponding to the extreme positions of the TM) are plotted in the corresponding colors.

Supplementary Movie 1 shows a radiography where the projections are post-gated into the different phases of movement: it enabled the visualization of the periodic movement of the TM without any prior 3D reconstructions. A slice in the 3D volume reconstructed at phase *p*_0_ of the movement is shown in Fig. 1a. The junction between the malleus and the TM was nicely revealed with the use of phase-contrast. The intensity profiles along a line perpendicular to the cross-section of the TM (yellow line) were extracted for each phase of movement. They are displayed as a 2D image in Fig. 1b, showing the sinusoidal displacement of the TM. The amplitude of displacement could be estimated as the shift between the two extreme positions of the TM: around 24 μm for an acoustic stimulation at 140 dB SPL. This corresponds to an amplitude of displacement within a 2D plane and at the specific location shown in Fig. 1a.

**Fig. 2:**
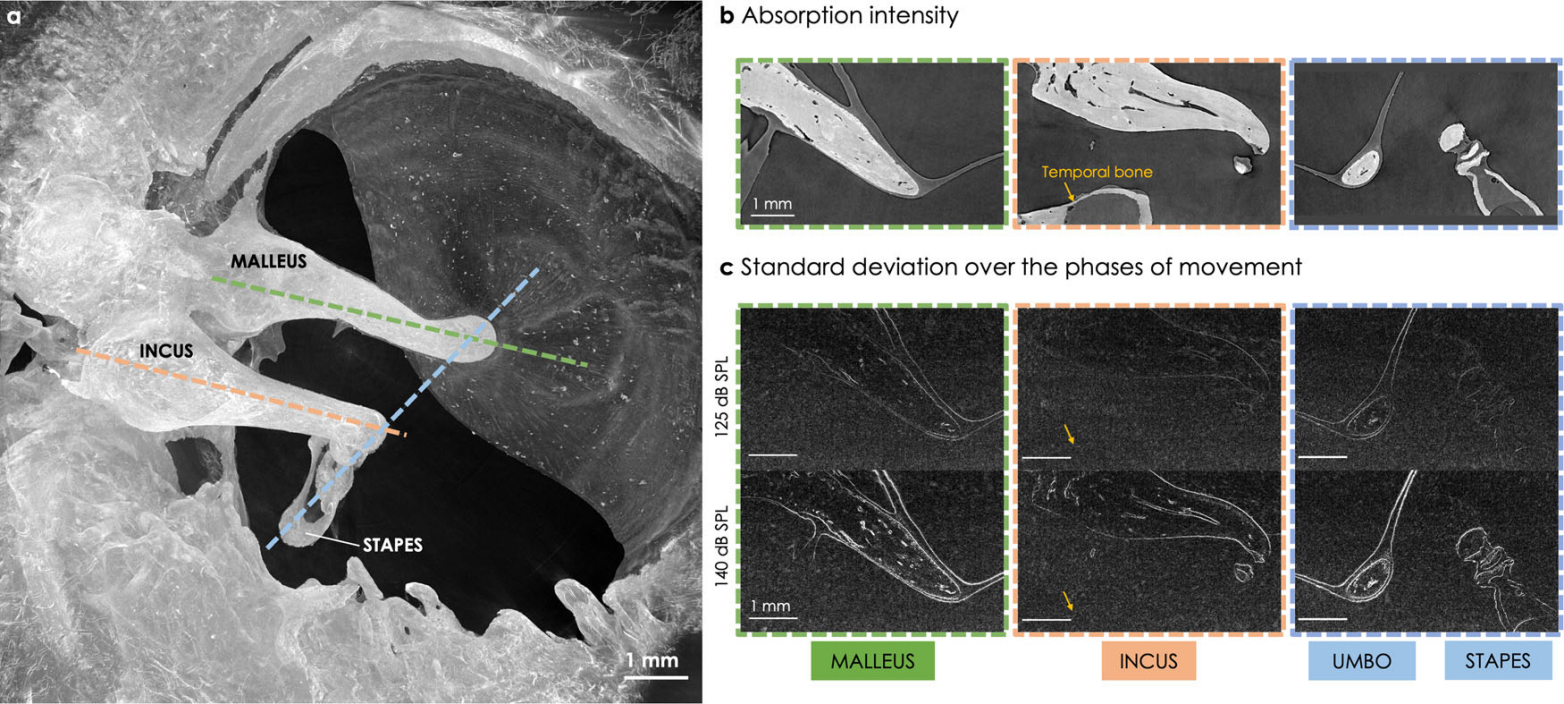
2D visualization of the ossicular motions in a fresh-frozen human specimen (*Fresh1*) stimulated at 127 Hz and 2 different sound pressure levels (SPLs). **a** The 3D volume containing the ossicular chain reconstructed at phase *p*_0_ is projected in 2D using a maximum intensity projection. **b** The 3D volume shown in **a** is vertically resliced along the 3 dashed lines shown in **a**, along the malleus (green), the incus (orange) and the stapes and umbo (blue). **c** The reslice shown in **b** is done in each 3D volume for all phases of movement. The ten resliced images of the same region are then projected in 2D with a standard deviation projection, that highlights the moving features. The yellow arrows point to the location of the temporal bone (static during the sound stimulation).

The ossicular motions were visualized after reconstruction of the static 3D volumes for each phase of movement. To that purpose, a 2D section through the 3D volumes was selected and compared between all phases of movement (see Supplementary Movie 2 to visualize the movement and Fig. 2b). More precisely, three sections were taken in a plane where the ossicular movement was assumed to be the largest: along the manubrium of the malleus, the long process of the incus, and the crus of the stapes (see the dashed lines in Fig. 2a). The standard deviation of the pixel gray value over the phases was plotted for every pixel of the 2D section (see Fig. 2c). It enabled the specific visualization of the moving features: the bright edges correspond to a stronger standard deviation due to the ossicular vibration over time, and their width and brightness give an indication of the amplitude of displacement, while a static feature exhibits a zero standard deviation. For example, the part of the temporal bone visible below the incus (yellow arrow in Fig. 2b) disappears completely after the standard deviation projection, confirming the absence of movement of the whole bone in comparison to the ossicles. As a proof of concept that the sound was well transmitted from the external auditory canal all the way to the inner ear, the stapes footplate movement inside the oval window - relative to the surrounding static cochlea - is shown in Supplementary Movie 3.

To overcome the loss of displacement due to the projection into a 2D plane, and to directly visualize the 3D motions of the ossicles, the reconstructed 4D datasets were imported in the Amira-Avizo software (Thermo Scientific Co., version 2020.3.1, see Materials and Methods). The three auditory ossicles could be segmented out of the complete 3D volume and displayed for different phases of movement. Supplementary Movie 4 loops over two segmentations of the ossicular chain, performed at the 2 extreme phases of movement, i.e., exhibiting the largest position difference.

With such a direct 3D visualization, one could think of manually selecting landmarks at different phases and computing the distance between them to extract the amplitudes of movement. However, considering the tiny movement of the ossicles, the accuracy of this approach is not satisfying as the error made in the manual selection of the landmarks is at least of the same order of magnitude than the amplitudes of displacement themselves. In addition, when going to more physiological stimulus conditions (lower sound pressure levels), the movements become even tinier: the TM movement in the 2D slice shown in Fig. 1a drops by a factor of 3 when going from 140 dB SPL to 125 dB SPL (from 24 μm to 8 μm). The ossicular motions become very difficult to identify, as seen in Fig. 2b where the moving features almost disappeared at 125 dB SPL. Consequently, a different analytical approach should be considered to quantify the motion.

### Extraction of the 3D ossicular motions at lower sound pressure levels

Subsequent to the pilot study of the first fresh-frozen sample, six other fresh-frozen human specimen (*Fresh3*, *Fresh7*, *Fresh8*, *B-Fresh1*, *B-Fresh2*, and *B-Fresh3*) were scanned under an acoustic stimulation at 128 Hz and two different sound pressure levels, 110 dB SPL and 120 dB SPL, closer to physiological conditions. As the bone absorption was high enough to maintain a satisfactory contrast for the ossicular chain, no phase retrieval algorithm was used to reconstruct the 3D volumes. That way, the system performance was kept at its maximum in terms of resolution (calculated with Fourier Ring Correlation as approximately 5 μm with a voxel size of 2.75 μm).

**Fig. 3:**
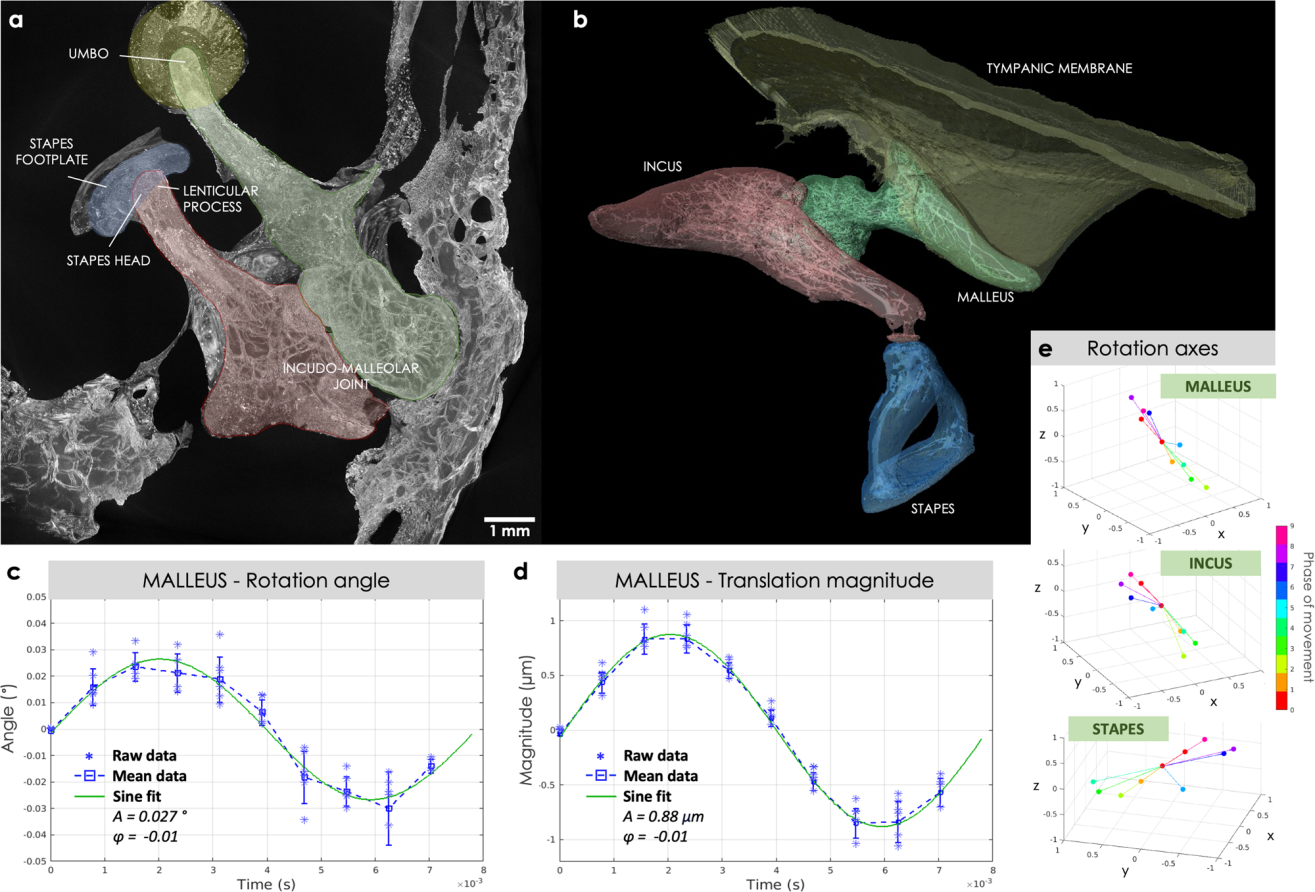
Extraction of the ossicular motions in a fresh-frozen human specimen (*B-Fresh3*) stimulated at 128 Hz and 120 dB sound pressure level. **a** Maximum intensity projection and **b** 3D visualization with segmentation of the static tomographic reconstruction at phase *p*_0_ of the movement, showing the tympanic membrane and the ossicular chain. The tympanic membrane, malleus, incus, and stapes are shown in yellow, green, red, and blue, respectively. **c** Rotation angles and **d** magnitudes of the translation vectors of the malleus as a function of time (one blue square per phase of movement). The raw data is shown in blue, with stars for individual data points, squares for the mean values and error bars for the standard deviations of the distribution across the *n* = 7 independent sub-volumes. **e** The rotation axes of the three ossicles are displayed for each phase of movement in rainbow colors. All the rotation axes are plotted from the same origin and the magnitude is scaled according to the rotation angle and normalized.

To be able to quantify the movement of the ossicles in 3D, at lower sound pressure levels down to 110 dB SPL, we developed a data analysis pipeline, extensively described in Materials and Methods, that used an image registration algorithm to compute the geometrical transformations of the ossicles over the different phases of movement. The pipeline assumed that each ossicle moved as a rigid body, i.e., the transformation of the ossicle consisted of a pure rotation followed by a translation, and it processed every ossicle independently (see Fig. 3a). For the two acoustic stimulation conditions (at 110 and 120 dB SPL), the translation vectors and the rotation matrices were extracted on the three ossicles of the six samples, for each of the 10 phases of movement.

As an example, Figure 3c, d shows respectively the rotation angles and the magnitudes of the translation vectors over time (the blue stars represents the phases of movement which are 0.78 ms apart from each other), extracted from the malleus of one fresh-frozen human specimen (*B-Fresh3*), while subjected to a 120-dB SPL acoustic stimulation. A sinusoidal variation over the movement cycle is found for both the rotation and the translation magnitudes. In addition to the rotation angles, the rotation axes are also available, providing the directions of the rotation axes over time. Figure 3**e** shows the rotation axes for the three ossicles as a function of the phase of movement. No dispersion in direction (all arrows overlapping) would mean a unique rotation axis over the full motion cycle, and would corroborate the current assumption of a rotation about a single axis, at low stimulus frequency. Here a small dispersion is observed, but a main direction is still detectable and computed by averaging all axes. The main directions of the rotation axis for the malleus and the incus are colinear. A similar small dispersion around a principal direction was noticed for all samples.

**Fig. 4:**
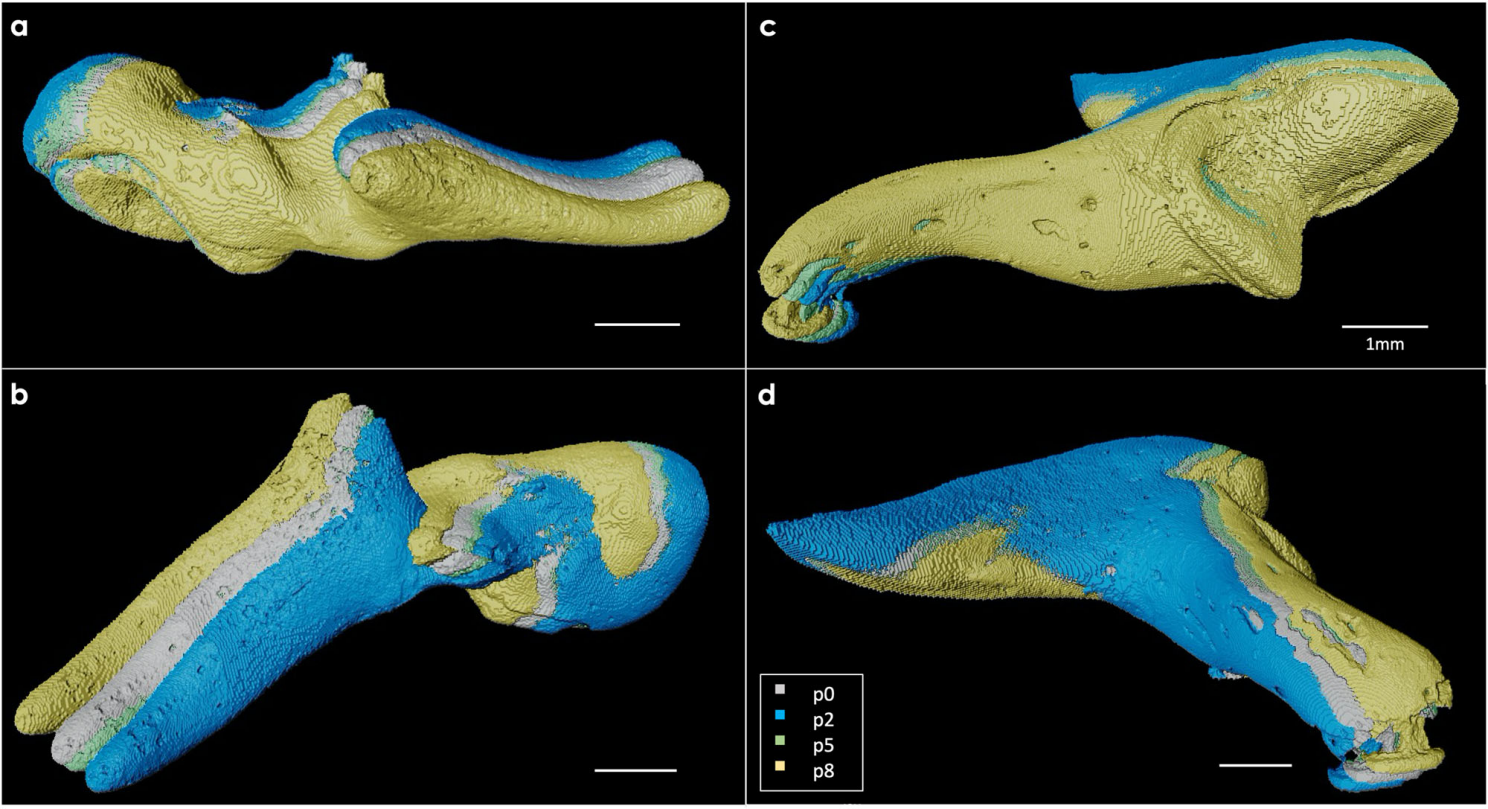
3D visualization of the malleus and incus motions in a fresh-frozen human specimen (*B-Fresh3*) stimulated at 128 Hz and 120 dB sound pressure level. **a** Cranial and **b** lateral view for the malleus, and **c** medial and **d** lateral view for the incus positions at 4 different phases of the movement: *p*_0_, *p*_2_, *p*_5_, and *p*_8_, shown in gray, blue, green, and yellow, respectively. The vibration amplitude is amplified x100 for visualization purpose.

**Fig. 5:**
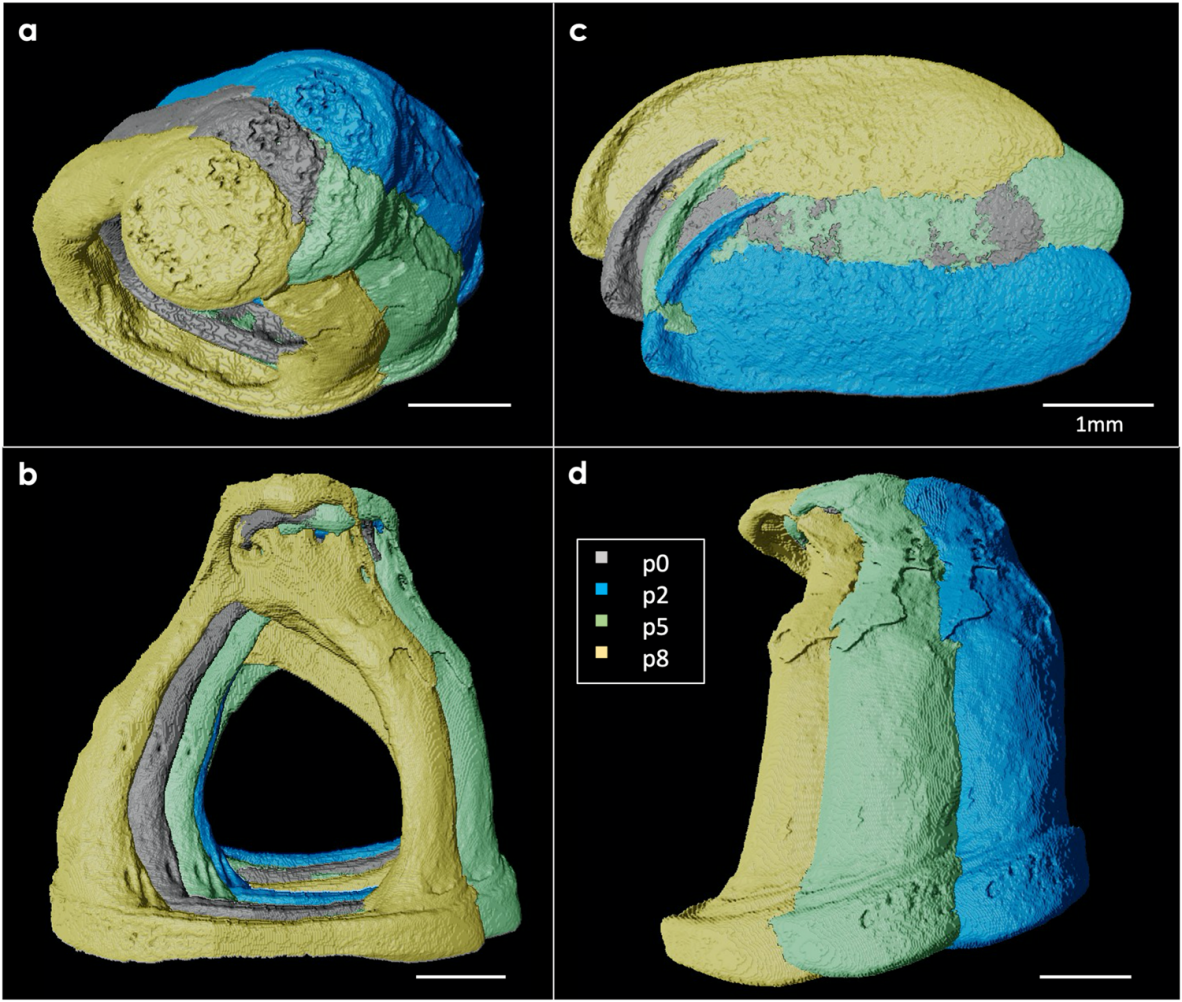
3D visualization of the stapes motions in a fresh-frozen human specimen (*B-Fresh3*) stimulated at 128 Hz and 120 dB sound pressure level. **a** Cranial, **b** lateral, **c** vestibular, and **d** posterior view of the stapes positions at 4 different phases of the movement: *p*_0_, *p*_2_, *p*_5_, and *p*_8_, displayed in gray, blue, green and yellow, respectively. The vibration amplitude is amplified x100 for visualization purpose.

These geometrical transformations could then be visualized in 3D. As a first step, the 3D volume at phase *p*_0_ of the movement was imported in the Amira-Avizo software, and the ossicular chain was segmented with three independent labels corresponding to the three ossicles. Figure 3d shows a 3D representation of the segmented tympanic membrane and ossicular chain of a fresh-frozen human specimen (*B-Fresh3*). Note that the cone-shaped structure of the tympanic membrane and its connection to the malleus are visible. The internal vascular systems of the ossicles is also highlighted with the 3D rendering. The second step was to amplify by a factor of 100 the transformations extracted beforehand for the three ossicles and to apply them to the corresponding segmented labels. A visualization of the periodic motion of the ossicular chain over time was obtained by repeating the last step for different phases of movement, and displaying all the transformed segmented objects simultaneously. As an example, visualization of the ossicular movement of a fresh-frozen human specimen (*B-Fresh3*) subjected to a 120-dB SPL acoustic stimulation is shown in Figs. 4 and 5. The three ossicles are shown in 4 different phases of motion, *p*_0_, *p*_2_, *p*_5_, and *p*_8_. Phases *p*_0_ and *p*_5_ (in gray and green) almost overlap, while phases *p*_2_ and *p*_8_ (in blue and yellow) are the two extreme positions of the movement, as expected from Fig. 3b-c. The periodic motion of the entire ossicular chain is also visible in Supplementary Movie 5.

**Fig. 6:**
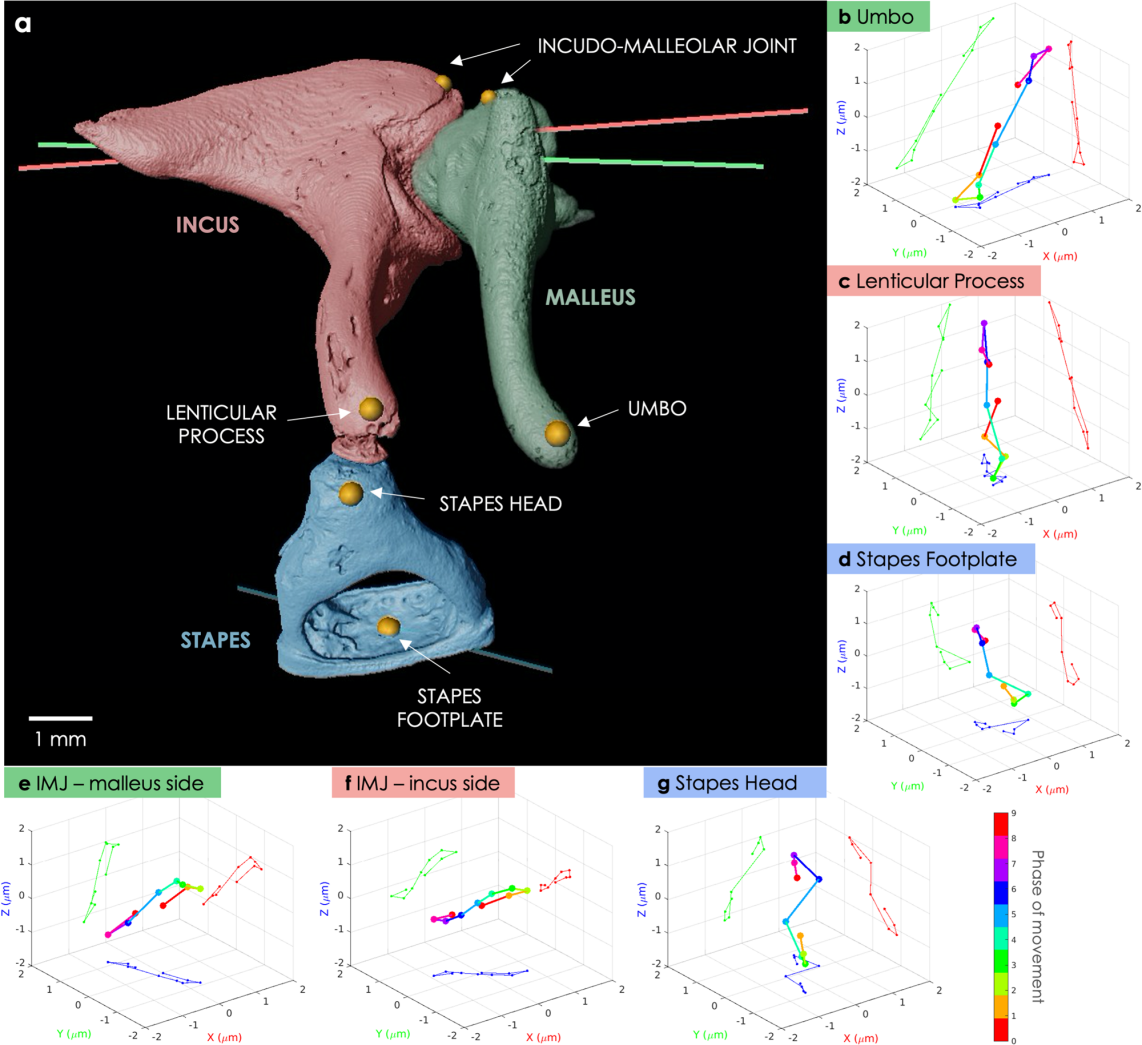
Displacement of different regions of interest within the ossicular chain in a fresh-frozen human specimen (*B-Fresh3*) stimulated at 128 Hz and 120 dB sound pressure level. **a** 3D visualization of the segmented ossicular chain at phase *p*_0_ of the movement. The six selected ROIs are marked with a golden dot and labeled: the stapes footplate, the stapes head, the lenticular process, the umbo and two points belonging to the incus and the malleus, respectively, near their common joint. The colored lines correspond to the principal axes of rotation of the malleus (green), incus (red), and stapes (blue). **b**–**g** Visualization of the 3D displacement of the ROIs, **a** the umbo, **b** the lenticular process, **c** the stapes footplate, **d** a point in the malleus near the incudo-malleolar joint, **e** a point in the incus near the incudo-malleolar joint and **f** the stapes head. Each color of the 3D rainbow plots corresponds to a phase of movement (from phase *p*_0_ - used as the origin (0,0,0) - to phase *p*_9_), and the 2D projections of the displacements are shown on the XY (blue), YZ (red), and ZX (green) planes.

The principal axis of rotation (averaged over the phases of motion) for *B-Fresh3* stimulated at 120 dB SPL is plotted for each ossicle in Fig. 6a. The rotation axes found for the malleus and incus correspond to their primary axis - perpendicular to the IM joint - and are consistent with a lever-like rotational motion about the IM joint. The axis of rotation found for the stapes has not been described before and is consistent with the shifted vibration displacement visible in Fig. 5.

### Displacement quantification of relevant regions in the ossicular chain

Having extracted the ossicular movement over time, we could quantify the displacement of any region of interest (ROI) within the ossicular chain. Six ROIs presenting a biomechanical interest and whose displacement velocities are usual measured with Laser Doppler Vibrometer were selected, two per ossicle: the umbo that connects the center of the tympanic membrane and the malleus, the center of the malleus head near the IM joint, the center of the incus body near the IM joint, the lenticular process at the tip of the long crus of the incus, the center of the stapes head and the center of the stapes footplate (see Fig. 6a).

Several points were selected within each ROI to cover its whole extend, and the geometrical transformation of the corresponding ossicle was applied to them in order to get their displacement for every phase of movement. A mean displacement was computed by averaging them. Figure 6b–g presents the 3D displacements over time of the ROIs in a fresh-frozen human specimen (*B-Fresh3*). The displacements of the ROIs are shown to be mostly sinusoidal along a single line. However, the stapes displacements show a 8-shape periodicity: slight lateral movements are revealed here, in addition to the expected uni-directional vibration.

The displacement norms were extracted for every phase of movement, given a sign corresponding to the displacement direction, and fitted with the following constrained sine function:

1 $$D\,(t)=A\left(\right.\sin (2\pi \,({{{{{{{\boldsymbol{f}}}}}}}}t+\phi ))$$

where ***f*** is the corresponding acoustic stimulation frequency, ***f*** = 128 Hz. The amplitude of displacement as well as the phase shift between the acoustic stimulus and the ROI motion were obtained from the fit parameters as *A* and *ϕ*, respectively.

**Fig. 7:**
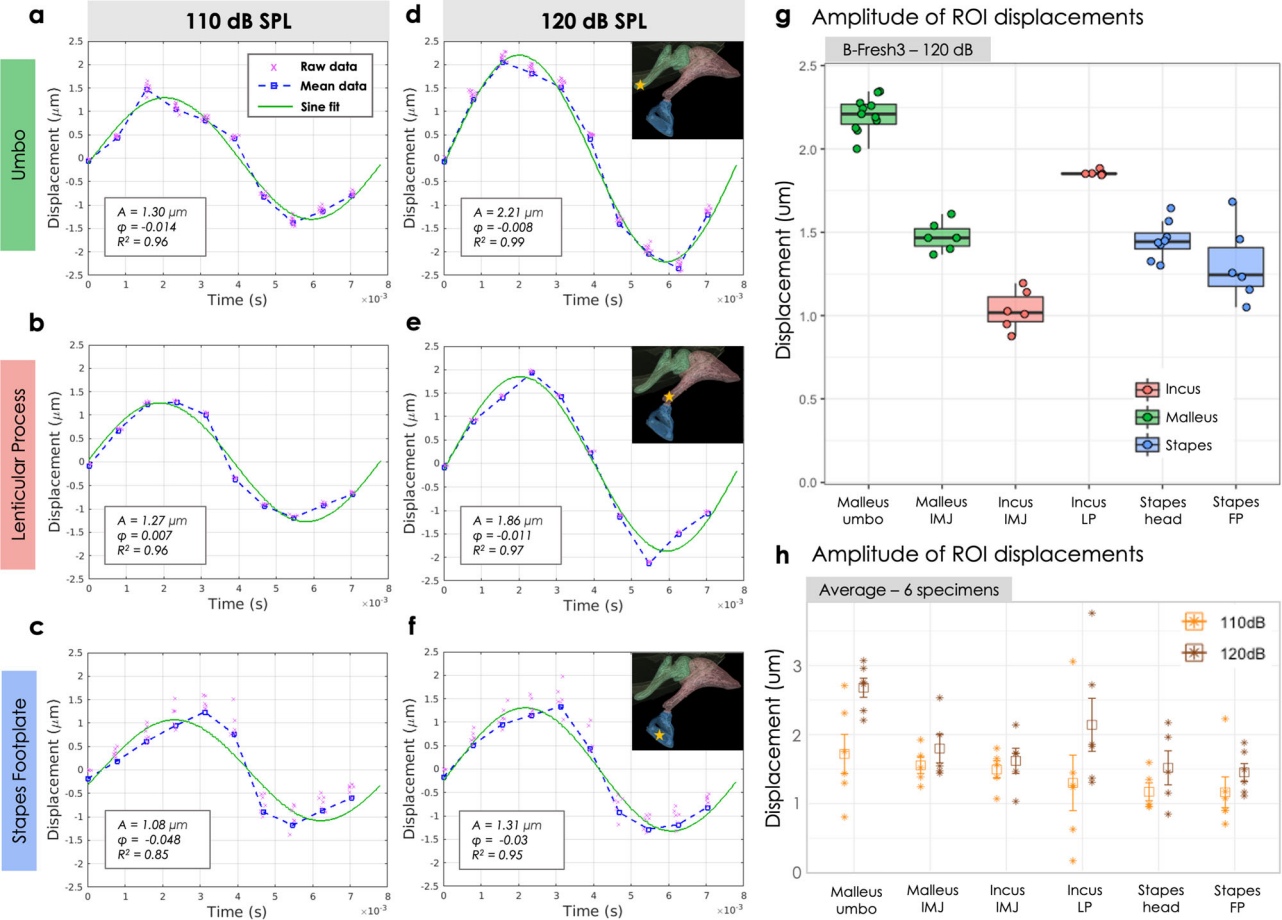
Amplitude of displacement of the regions of interest (ROIs) within the ossicular chain. **a**–**f** The magnitudes of the displacement vectors are shown over time for a fresh-frozen human specimen (*B-Fresh3*) stimulated at **a**–**c** 110 dB SPL and at **d**–**f** 120 dB SPL, for **a**, **d** the umbo, **b**, **e** the lenticular process and **c**, **f** the stapes footplate. The raw data (dashed blue line) are fitted with a sine (solid green line). The distribution across the *n* = (11, 5, 6) independently selected points (respectively for the umbo, lenticular process and footplate) is shown: blue squares for mean values and pink crosses for individual data points. **g** The amplitudes of displacement of *B-Fresh3* stimulated at 120 dB SPL are plotted as a function of the ROIs. Each individual point correspond to a manual selection of a point within the ROI. **h** The amplitudes of displacement averaged over the 6 samples are shown as a function of the ROIs, for the two different stimulation sound pressure levels, 110 dB SPL (orange) and 120 dB SPL (brown). Individual data points are shown as stars, mean values as squares and the error bars represent the standard deviations of the distributions across the *n* = 6 independent samples.

Figure 7a–f displays the displacement norms of three ROIs of the human fresh-frozen specimens *B-Fresh3*, at the two SPL used. The sine fits are shown as green solid lines, and the goodness of fit of the sine model is measured via the coefficient of determination *R*^2^.

In Fig. 7g, the amplitudes of displacement 2*A* are plotted as a function of the ROIs. For the 2 long-shape ossicles - the malleus and the incus - the ROIs located at the extremity of the arms exhibit bigger displacement as the ROIs near the IM joint, in line with a movement of mostly pure rotation about an axis crossing perpendicularly the IM joint. For the stapes, the 2 ROIs taken from the head and the footplate do not show significant difference, reinforcing the assumption of a translation movement mainly along one principal axis. In addition, a decrease in the displacement amplitude is noted when the measured point goes further away from the tympanic membrane along the ossicular chain: the umbo exhibits the highest displacement, followed by the lenticular process and finally the stapes footplate. Moreover, the movements obtained at a 110-dB SPL and a 120-dB SPL acoustic stimulation were compared. With a 10-dB SPL increase, the displacement is amplified by an average factor of 1.7, 1.5, and 1.2 for the umbo, the lenticular process, and the stapes footplate, respectively.

A similar plot where each point is an average over the six specimen is proposed in Fig. 7h. Although the inter-sample dispersion is quite high, the same tendency of a displacement dampening along the ossicular chain is revealed, both at 110 dB SPL and at 120 dB SPL, with a displacement amplification of the order of 1.5 between the two stimulation SPL.

## Discussion

In this paper, the use of dynamic synchrotron-based X-ray microtomography to visualize the micromotions of the in-situ intact human middle ear under acoustic stimulation is reported. It characterizes the motions of the ossicular chain down to the stapes, the tiniest bone in the human body whose footplate has an approximate size of 2.8 × 1.37 mm^2^ ^23^, and whose motion visualization has always been a technical challenge. This is a novel and innovative approach to study the biomechanics of the human middle ear, as it provides a 4D visualization of the complete moving system. Other standard techniques, like Laser Doppler Vibrometry, generally probe only one dimension of motion and are invasive, although they do have the advantage to resolve smaller motion amplitudes. The contrast enhanced with the partial coherence of synchrotron-based X-ray beam allowed us to conduct measurements on fresh-frozen samples in their natural state. We could investigate the motions of the middle ear stimulated at 128 Hz and with sound pressure levels in the physiological range. The dynamic microtomography acquisitions were successfully achieved at a large FOV of the camera with a total scan time of only 20 seconds per acquisition. Maintaining a short scan time is crucial to limit the total dose of X-ray radiation that is absorbed, especially while working with a high dose rate. Here, the dose rate is assumed to be similar to the one estimated previously at 300 Gy.s^−1^ ^21^. Four-dimensional reconstructions of the vibrating auditory chain were obtained with a voxel size of 2.75 μm (and a corresponding true spatial resolution of 5 μm) and an effective temporal resolution of 2 kHz. An acoustic stimulation may be performed at higher frequencies, but at the cost of a shorter exposure time, leading to a reduction of the image quality, and of a smaller amplitude of movement below the accessible resolution. Resolution limitations may be overcome in the future, with the recent acquisition by TOMCAT of a high-magnification microscope and the upgrade of the Swiss Light Source, extending the possibility of ultra-fast dynamic investigations with synchrotron imaging.

In addition to the visualization possibility, this work also presents a high-throughput pipeline to extract the geometrical transformations of the individual ossicles. It enables the amplification of the transformations for visualization purposes on the one hand, and on the other hand the computation of quantitative biomechanical parameters of the ossicular chain, such as the rotation axis of the ossicles, their rotation angle and their absolute translation (magnitude and direction). For each ossicle, the amplitudes of displacement of six specific regions of interest on the ossicular chain were computed and averaged over six fresh-frozen samples. Our numerical approach could quantify shifts even below the voxel size, down to 0.5 μm where the limits reached both in spatial resolution and image quality prevent the detection of movement. The ossicular response over time was shown to be essentially sinusoidal, and showed amplitudes of displacement in line with previous measurements with laser Doppler vibrometry^24^. The movements of the regions of interest were tracked in 3D and revealed a 8-shape vibration, not constrained along a one-dimensional line, proving the importance of a 3D characterization.

The 4D visualization of one sample is shown and confirms a lever-like motion for the malleus and the incus, i.e., a rotational motion about the primary axis perpendicular to the IM joint axis. On the other hand, the detected stapes motion for this specific sample challenges the idea of a pure piston-like medial motion: although a medial motion is still detected, a larger infero-posterior motion is also present. However, we did not discard it as clinically irrelevant, as this type of rocking stapes motion has already been observed in the literature^25^. In addition, the motions differ from one sample to another, and further investigations on the stapes movement with exhaustive motion analyses taking into account anatomical and patient-related features would be crucial in the future to understand the complex stapes motion that has not been completely described in 3D so far.

The computation of the quantitative biomechanical parameters could, however, be refined without assuming rigid-body motions, but including the eventuality of heterogeneous movements within an ossicle, e.g., due to a variation of its porosity. That could explain the slight dispersion of the parameter values computed from different sub-volumes of the same ossicle (see Supplementary Fig. 2c). Indeed, although the rigid-body assumption is mostly used in the literature, as the ossicles are bony material, it stays an approximation for biological tissue. For example, works from Decraemer et al. conducted on cat middle ears, showed that the manubrium could go through elliptical motions as well as some bending^26,27^. However, this has been observed for a regime of frequencies above 2.5 kHz - much higher than the one we are using in our study (128 Hz), where the rigid body approximation should still be accurate.

Novel non-rigid approaches will be considered in the future, to be applied as well to the non-rigid tympanic membrane in addition to the ossicles. Furthermore, non-linearities that could emerge from the anatomy of the middle ear itself, depending on the individual samples, at specific acoustic conditions, are not taken into account here^28,29^. The sine behavior of the displacement amplitudes allows us to rule out the presence of non-linear responses at 128 Hz, but further investigations at higher frequencies are on-going to identify potential non-linear responses.

Future implications of this work include the analysis of conductive hearing loss and investigation of the impact of middle ear pathologies on sound transmission. Specifically, a disease and the efficiency of its surgical treatment could be assessed by computing the biomechanical parameters of pathological vs. treated samples imaged with dynamic synchrotron-based X-ray microtomography. Furthermore, it is expected that the present work will have an impact beyond the imaging and clinical communities. Extending the fundamental understanding of the biomechanics and the anatomy of the human middle ear through dynamic high-resolution images will strongly benefit the finite elements community, which aims to build comprehensive and reliable 4D models of the human middle ear.

## Materials and methods

### Samples

The study protocol was approved by the local ethical committee of Bern (Kantonale Ethikkommission Bern, KEK-BE 2016-00887) and the local ethical committee of the Paul Scherrer Institute (Ethikkommission Nordwest-und Zentralschweiz, 2017-00805), as well as the Mass General Brigham Institutional Review Board (#2022P001306).

Seven human fresh-frozen temporal bones were obtained from anonymous body donors: four from the institute of pathology in Bern, Switzerland (*Fresh1*, *Fresh3*, *Fresh7*, and *Fresh8*) and three from the Eaton Peabody Laboratories, Mass Eye and Ear, Boston, MA, USA (*B-Fresh1*, *B-Fresh2*, and *B-Fresh3*). The naming of the samples is the one used during the experiment and stored as such in the Petabyte Archive System of PSI (a tape based long-term storage system located at the Swiss National Computing Center CSCS in Lugano, Switzerland). It is kept as such in this paper to be in line with the FAIR principles: each dataset is uniquely identified with a globally unique persistent identifier (PID), and freely accessible with the same naming.

The seven samples were prepared identically in Bern. They were lesion free and without structural pathologies. The auditory system was kept intact while removing as much bony tissue as possible to keep external X-ray absorption to a minimum. Laterally, the concha was removed, conserving the bony and cartilaginous external auditory canal. Consecutively, the bony external auditory canal was thinned to 1 mm bone thickness using a surgical burr. Posteriorly and superiorly, the air cells of the mastoid portion were removed in the same way. The tegmen tympani and the antrum, the facial nerve, and the middle ear cavity were skeletonized. Inferiorly, the soft tissue was removed including the internal carotid artery, the jugular bulb, and the cartilaginous insertion of the Eustachian tube. Medially, the petrous part of the temporal bone was removed until the bony capsule of the labyrinth. The semicircular canals and the internal auditory canal were skeletonized. In the end, the samples were 50 mm in height and 24 mm in diameter, but the auditory system was kept intact and complete, with an intact external auditory canal, tympanic membrane, middle ear, and inner ear.

During the imaging acquisition, each specimen was placed in a custom-made cylindrical holder with a diameter of 25 mm, which was screwed onto the rotation stage. They were wrapped in neuro-patties soaked in a sterile saline solution to prevent the samples from drying out during the acquisition. The top of the sample holder was sealed with a plastic film.

### Sound stimulation and calibration

**Fig. 8:**
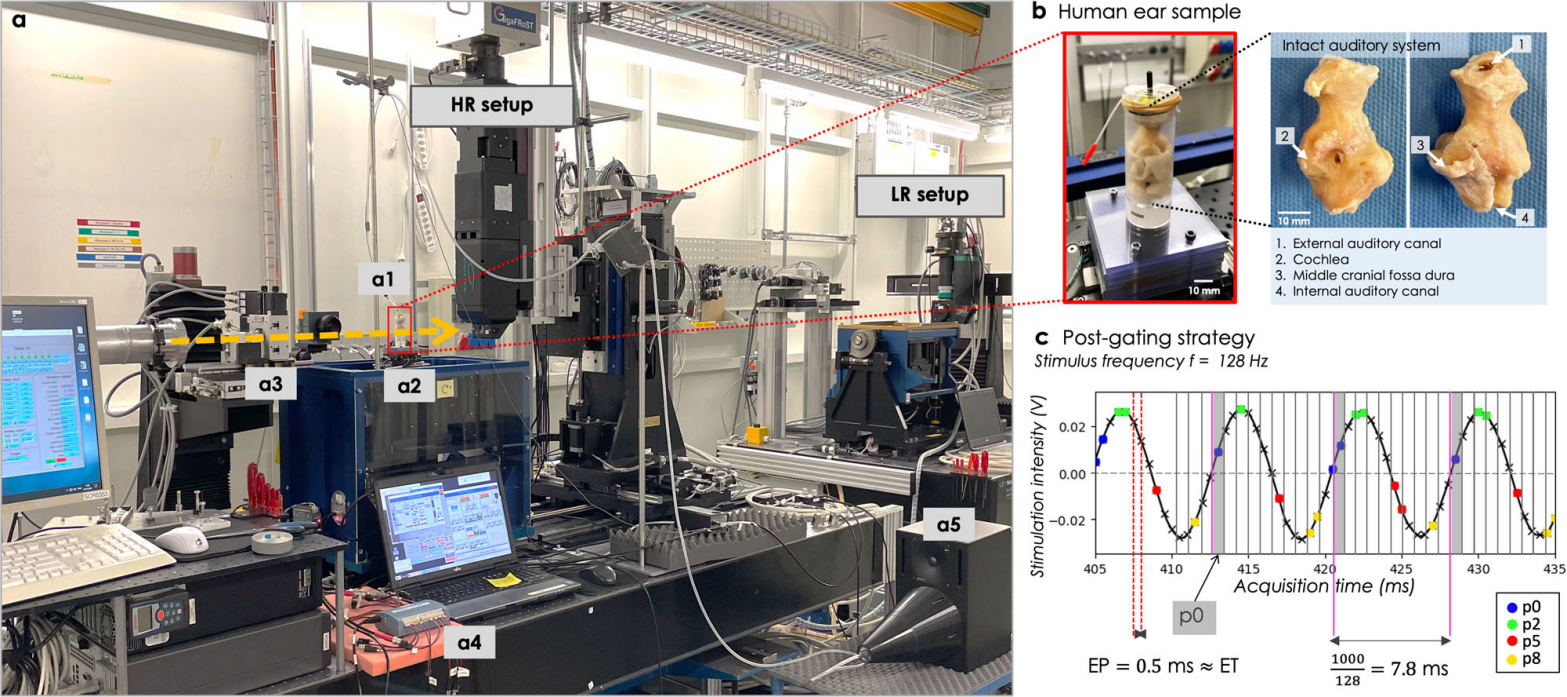
Image acquisition and post-gating. **a** Experimental setup implemented at the TOMCAT beamline. The sample in its holder **a1** is placed on the sample manipulator **a2** and illuminated with the X-ray beam coming from the left (yellow arrow). A shutter and slits system **a3** protect the sample between acquisitions and adjust the beam size to the corresponding field of view of the low-resolution (LR) setup or the high-resolution (HR) setup. The microscope tower of the HR setup can be translated to let the X-ray beam reach the LR setup. The sound wave is produced by a signal generator **a4** connected to a subwoofer **a5**. The sinusoidal signal generated is systematically recorded, as shown in **c** for a sound stimulation at 120 dB and 128 Hz. **b** Zoom-in on the sample holder: the black cylinder is used to plug the silicon tube carrying sound stimulation, and the red-headed tube is used to plug a probe microphone for sound calibration. The full peripheral auditory system of the sample is intact. **c** The post-gating processing retrospectively associates each image with its corresponding phase of motion, among the 10 phases used to decompose one period of the recorded signal (gray vertical lines, each period starting at the pink line). As examples, the yellow, blue, green, and red points show the images post-gated into the motion phases *p*_1_, *p*_3_, *p*_5_ and *p*_8_, respectively.

The proof of concept of the sound stimulation protocol was done in one fresh-frozen human specimen (*Fresh1*), stimulated at one pure tone frequency (127 Hz). Two constant sound pressure levels (SPL in dB) applied inside the auditory canal were chosen for these tests: first a high intensity of 140 dB to guarantee a visible movement, and then a lower intenity of 125 dB to get closer to physiological sound stimulation. Sound stimulation of the other six samples was performed at 128 Hz and at two lower SPL (110 and 120 dB). The stimuli were delivered to the sound source via a sine wave generator (MeasComp USB daq Module MC1608 USB-1608G SKU: 6069-410-059). A silicon tube connected the sound stimulation unit to an ear plug fixed in the external auditory canal (see Fig. 8a). A custom-made sound stimulation unit was used, consisting of a subwoofer as sound source coupled with a inversely mounted horn^30,31^.

Before scanning each sample, the sound stimulation was calibrated by measuring the exact SPL applied in the auditory canal with a clinical probe microphone (ER7C, Etymotic Research). The probe microphone was removed from the experimental hutch during acquisitions to prevent any damage due to humidity coming from the sample, twisting cables during tomography, or radiation damage with X-rays. These calibrations were reproducible and guaranteed the homogeneity of stimulations across samples.

### Image acquisition

Dynamic synchrotron-based X-ray phase-contrast microtomography was performed at the TOMCAT beamline (X02DA) of the Swiss Light Source (Paul Scherrer Institute, Switzerland). Due to the size of the samples, a multi-scale approach was carried out as follows: a low-resolution (LR) setup was used to acquire overview scans of the sample, and then high-resolution (HR) scans of the middle ear were performed locally (see Fig. 8a). An in-house developed Fiji plugin, using as an input the 3D reconstructed LR dataset, provided the spatial coordinates of the regions of interest (namely the tympanic membrane and the ossicular chain) to be collected with the HR setup (more details on the multi-scale protocol can be found in Dejea et al.^32^). The LR overview scans covered a field-of-view (FOV) of about 29 × 12.5 mm^2^, using the so-called half-acquisition method, i.e., using 360^∘^ rotation instead of 180^∘^ in a standard tomography acquisition. The setup consisted of a PCO 5.5 Edge camera combined with a 1:1 microscope 3 m away from the sample, which gave an effective pixel size of 5.8 μm. The scan time was adjusted to reduce the dose to a minimum (30 ms exposure time, 1000 projections over 360^∘^). The dynamic HR acquisitions were performed with a custom-made in-house fast read-out system (the GigaFRoST camera^19^), a LuAg:Ce 150-μm-thick scintillator, and a 4x magnification high numerical aperture macroscope from Optique Peter^33^, at a propagation distance of 250 mm, achieving an effective pixel size of 2.75 μm (FOV about 11 × 3.3 mm^2^ using the half-acquisition method).

The exposure period (i.e., the time between two image acquisitions) was set to 0.5 ms, i.e., a frame rate of 2 kHz. This is the maximum frame rate achievable at the maximum FOV possible with the read-out system GigaFRoST before saturation of the data transfer.

As the middle ear motion is assumed to be a periodic vibration with the same frequency as the sound stimulation, the timescale of one motion cycle is much shorter than the time needed to obtain a complete set of angular projections (several thousands of images needed for a tomography acquisition). Consequently, the dynamic tomograms were built-up by acquiring a substantial number of projections over multiple and consecutive motion cycles, while the rotation stage slowly rotated. In total for one scan, 40,000 projections were acquired over a single 360^∘^ rotation of the sample. The exposure time (i.e., the effective time of photon collection) was set to 0.495, not to exceed one tenth of the sound stimulation period to not blur the image because of the movement.

A polychromatic beam was used for the LR and HR acquisitions. For the HR acquisitions, it was filtered with 5 mm of Sigradur and 4 mm of glass, resulting in an average energy of 24 keV. For the LR acquisitions, an additional 15-mm Sigradur and 75-μm Molybdean filters were used to further reduce the dose on the sample.

### Post-gating reconstruction

Two signals were collected during the image acquisition: the sinusoidal signal (or gating signal) transmitted from the signal generator to the sound unit and the camera exposure signal giving the exact time of each image acquisition. These two signals allowed to associate each image with a specific phase of the sine stimulation, corresponding to a specific phase of the vibration of the middle ear. The gating signal period was decomposed into ten different time windows called phases *p*_*j*_, with *p*_0_ being the reference phase taken at the ascending zero-crossing point of the sinusoidal curve (see Fig. 8(b1)). A post-gating algorithm was applied to the 40,000 raw projections to sort them into the correct phases and build ten post-gated tomograms of approximately 4000 projections. These 4000 projections were evenly distributed over the full 360^∘^ rotation of the sample, so that each post-gated tomogram allowed to reconstruct in 3D each specific phase of the middle ear movement cycle.

To correct for the X-ray beam inhomogeneities and dark current of the camera, the projections were first dark- and flat-field corrected. The sinograms were then computed for each set of projections, and the reconstructions were achieved using the filtered back-projection Gridrec algorithm^34^ and the Sarepy algorithm for ring removal^35^. Due to the coherence of the synchrotron X-ray beam, the X-ray images contained phase-contrast even when phase retrieval filters were not used for the 3D reconstructions. This phenomena was exploited further to increase the contrast-to-noise ratio (CNR) in the static measurements and the 2D dynamic measurements on the first specimen (*Fresh1*), thanks to a phase retrieval algorithm applied before the reconstruction (Paganin phase retrieval method with *δ* = 1e-7 and *β* = 1e-9)^36^.

### Data analysis: volume registration

To quantify the movement of the ossicular chain during sound stimulation, the ossicles were assumed to behave like independent rigid bodies. Under this assumption, their 3D motion over time is described by rigid transformations (Euclidean isometries), composed of a rotation followed by a translation, and all the points belonging to the same ossicle undergo the same transformations. Consequently, the analysis of only a sub-volume (SV) of an ossicle is enough to extract the transformation of the entire ossicle.

The middle ear movement cycle was decomposed into ten different time windows called phases *p*_*j*_. The intensity-based registration algorithm *imregtform* from Matlab was used to register in 3D the SV of an ossicle taken at phase *p*_0_ with the same SV taken at the other phases *p*_*j*_, with *j* ∈ [[1, 9]]., and estimate the geometrical transformation bridging the two phases. This type of algorithm does not require any segmentation nor manual landmark placement and effectively exploits all available information by use of the unaltered intensity of all image pixels, allowing in addition sub-voxel registration^37^.

The registration allowed us to compute the transformation matrix *T*_*j*_, from which a rotation matrix and a translation vector were extracted. The rotation matrix was then converted according to the Euler axis-angle formalism to get a unit vector indicating the direction of the axis of rotation and an angle describing the magnitude of the rotation about the axis. At the end, for every phase *p*_*j*_, a translation vector, a rotation axis vector, and a magnitude of rotation describing the transformation from phase *p*_0_ to phase *p*_*j*_ was computed for each ossicle.

The registration algorithm was parameterized to show optimal performance for most of our data with a Mattes mutual information metric and a regular step gradient descent optimizer, a maximum number of iterations, and minimum step length set to respectively 1000 and 10^−5^, three pyramid levels and a rigid geometrical transformation type.

### Data analysis: transformation averaging over the sub-volumes

In practice, to compromise between measurement reliability and computational time, 4 to 8 sub-volumes (SVs) per ossicle were analyzed. These SVs were manually selected with Fiji on the 3D data stack at phase *p*_0_ (see Supplementary Fig. 2a), and their coordinates were saved on a text file. Each SV was encompassed entirely within one ossicle and comprised no other features like ligament or static bone parts. The transformations of all SVs were averaged to get one mean transformation per phase for each ossicle. As the sub-volumes were not expressed from the same origin, the averaging followed a thorough procedure given below.

The spatial position of a specific point in an ossicle taken at phase *p*_*j*_ and expressed in a reference frame with origin *O*^(*i*)^ is given by the vector ${\vec{{x}_{j}}}^{(i)}$:

2 $${\vec{{x}_{j}}}^{(i)}={R}_{j}^{(i)}{\vec{{x}_{0}}}^{(i)}+{\vec{{t}_{j}}}^{(i)}$$

where *R*^(*i*)^ and ${\vec{t}}^{\,(i)}$ are, respectively, the rotation matrix and the translation vector describing the rigid transformation of the ossicle, expressed from the origin *O*^(*i*)^, center of the *i*^*th*^ SV.

The origin of the first selected SV *O*^(0)^ was chosen as the global origin for the ossicle, and the transformation of each SV was then re-expressed in the global coordinate frame, according to the unicity of an affine transformation (see Supplementary Fig. 2b):

3 $${R}_{j}^{(0)}=\,	{R}_{j}^{(i)}\\ {\vec{{t}_{j}}}^{(0)}=\,	{\vec{{t}_{j}}}^{(i)}+({R}_{j}^{(i)}-I)\vec{b}$$

where $\vec{b}=({O}^{(0)}-{O}^{(i)})$ is the difference vector between the local origin *O*^(*i*)^ of the SV and the global origin.

A mean transformation for every phase was then obtained by averaging the transformations of all SVs for a particular ossicle. More specifically, the mean translation vector, the mean rotation axis vector, and the mean magnitude of rotation could be calculated component-wise for every phase.

Based on the assumption of a rigid body motion, all SVs of a same ossicle should exhibit the same transformations. However, the performances of the *imregtform* algorithm could vary across the SVs as we dealt with small motions below the voxel size, especially for SVs selected close to the rotation axis where the displacement is reduced. Localized poor contrast to noise ratio or ring artifacts subsisting after post-processing could also affect the performances. Consequently, the transformation estimations for all SVs were compared, and SVs showing not sensible values were discarded to enable averaging over reliable estimations. An unbiased way to perform the latter was to fit the distribution of the seven parameters (translation and rotation vector components and rotation angle) with Gaussian distributions and use the distance to the normal mode *μ* as a sensible criterion. Specifically, the SVs showing values deviating from *μ* by more than two times the standard deviation were discarded. The values five times higher than the median value of the original distribution were also excluded: such high values were computed when the *imregtform* algorithm failed to converge.

### Data analysis: displacement calculations

Once the mean transformations of the three ossicles were obtained for all phases *p*_*j*_, the sinusoidal displacement of any region of interest (ROI) belonging to an ossicle could be computed, as the norms of the transformations applied to this point. Six ROIs, two per ossicle, were manually selected with Fiji for each sample on the reconstructed data stack taken at phase *p*_0_. To evaluate the precision of the displacement computation relative to a manual selection of the ROI, different points close to each other were picked to compute a standard deviation of the displacement estimations.

To make sure the transformations extracted corresponded to the vibrations of the stimulated ossicles, and not to the vibrations of the whole sample within the sample holder, we applied the pipeline on the temporal bone surrounding the middle ear (see Supplementary Fig. 3) and could verify that the translation magnitudes and the rotation angles were below respectively 0.2 μm and 0.002^∘^, leading to a maximum displacement magnitude of 0.3 μm. These values set the corresponding noise limit of our analyses.

### Statistics and reproducibility

A total of seven fresh-frozen human temporal bones were included in the study, representing the maximum sample size possible given the limited availability of temporal bones from body donors. One specimen was allocated for initial testing and proof-of-principle experiments. The same experiment was then repeated on the remaining six samples. To assess the general movement of the ossicles, we computed the average geometrical transformations across multiple sub-volumes (ranging from 5 to 7) selected within the ossicles. For the analysis of displacement in specific regions of interest, we calculated the average amplitudes of displacement across several points (ranging from 5 to 11) within those regions. Additionally, we averaged the amplitudes of displacement across all specimens (*n* = 6). While the sample size was sufficiently large to identify trends and understand inter-sample dispersion, it limited our ability to conduct further statistical tests.

### 3D and 4D visualization

The Amira-Avizo software (Thermo Scientific Co., version 2020.3.1) with the XImagePAQ - Advanced Image processing and quantification extension was used for the 3D and 4D visualization of the datasets. The software has previously proven its capability of handling big data^10^. With the extension, we can profit from additional advanced image processing and quantification tools, e.g., the watershed segmentation tool.

Because image files in the standard tiff file format are limited to 4 GB, the reconstructed tiff files were converted to the AmiraMesh file format (.am), which is the native file format of Amira. For the 3D visualization of the middle ear (see Fig. 3), the five sub-scans containing the ossicular chain and the tympanic membrane were loaded into Amira and registered one by one with the *Register Images* module. The mutual information metric was chosen as the similarity measure^38–40^. The merge module finally allowed us to end up with only one image containing the five sub-scans and the entire ossicular chain. In the Segmentation Editor, the watershed segmentation^41^ was initiated by placing markers on every 50*th* slides in the region of the stapes and every 100th in the remaining area. The stapes, the incus, the malleus, and the tympanic membrane were segmented the same way.

### Reporting summary

Further information on research design is available in the Nature Portfolio Reporting Summary linked to this article.

### Supplementary information

Supplementary Information

Description of Additional Supplementary Files

Supplementary Movie 1

Supplementary Movie 2

Supplementary Movie 3

Supplementary Movie 4

Supplementary Movie 5

Supplementary Data 1

Reporting Summary

## Data Availability

The raw data supporting the conclusions of this article have been archived and published in agreement with the FAIR principles, on the SciCat Data Catalog of PSI. They are openly available via a DOI: 10.16907/e4ae0d62-c7a1-4743-8d46-207e431be4ac. The derived measurements underlying the graphs in the manuscript are provided in Supplementary Data 1.

## Code availability

The custom Python and Matlab codes supporting the analyses shown in this article are available upon request.
