# Supplementary material for: The human middle ear in motion: 3D visualization and quantification using dynamic synchrotron-based X-ray imaging
Source: Commun Biol. 2024 Feb 7;7:157. doi: 10.1038/s42003-023-05738-6 (PMC10850498; doi:10.1038/s42003-023-05738-6)
Supplement: Supplementary file 1 — Supplementary Information [file 42003_2023_5738_MOESM1_ESM.pdf]

# The human middle ear in motion: 3D visualization and quantification using dynamic synchrotron-based X-ray imaging

Margaux Schmeltz [*,1,†], Aleksandra Ivanovic [1,2,3,†], Christian M. Schlepütz [1], Wilhelm Wimmer [2,4], Aaron K. Remenschneider[5], Marco Caversaccio[2,3], Marco Stampanoni [1,6], Lukas Anschuetz [2,3,‡], and Anne Bonnin [1,‡]

[1]Paul Scherrer Institute, Swiss Light Source, Villigen, Switzerland

[2]Department of Otorhinolaryngology, Head and Neck Surgery, Inselspital, Bern University Hospital, Bern, Switzerland

[3]Hearing Research Laboratory, ARTORG Center for Biomedical Engineering Research, University of Bern, Bern, Switzerland

[4]TUM School of Medicine, Klinikum rechts der Isar, Department of Otorhinolaryngology, Munich, Germany

[5]Department of Otolaryngology, Head and Neck Surgery, Mass. Eye and Ear, Boston Children Hospital, Harvard Medical School, Boston, USA

[6]Institute for Biomedical Engineering, University and ETH Zürich, Zurich, Switzerland

[†]These authors contributed equally.

[‡]These authors jointly supervised this work.

## Supplementary Information

### Supplementary Figures

[*]margaux.schmeltz@psi.ch

Supplementary Figure 1: **2D slices of a 3D stack of a fresh-frozen human specimen (*Fresh1*) reconstructed with phase-contrast.**

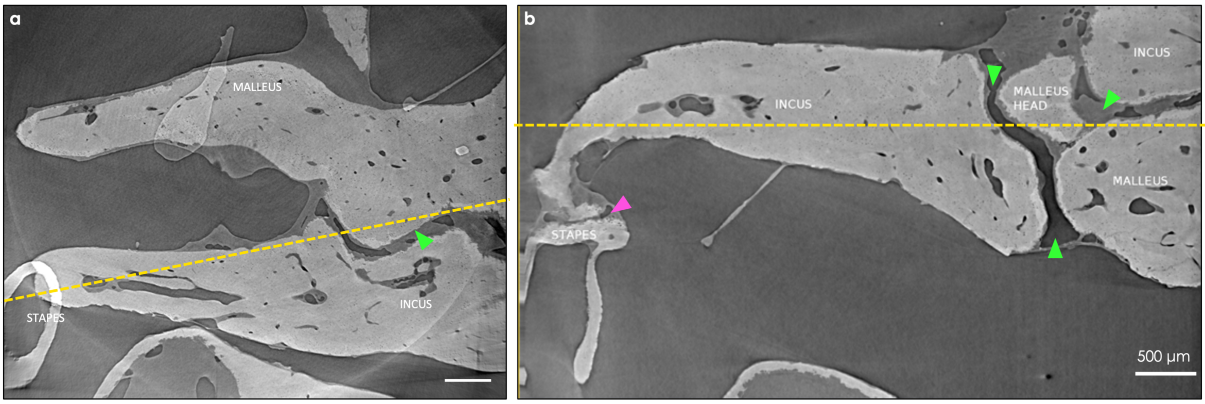


**a** Top-view and **b** lateral view of the stack resliced along the yellow line in **a**. The green and pink arrows respectively point at the incudomalleolar joint and the incudostapedial joint.

Supplementary Figure 2: **Transformation extraction from sub-volumes.**

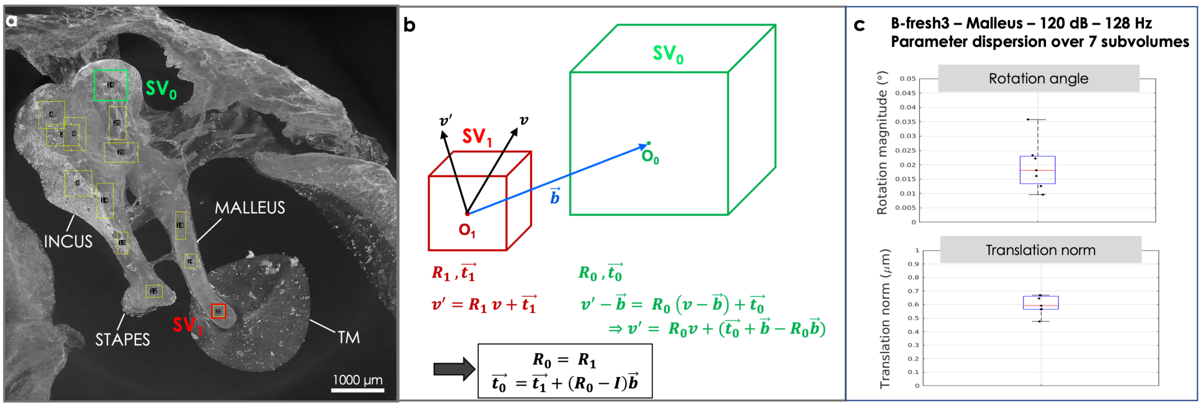


**a** 3D rendering of a fresh-frozen human specimen (*B-fresh1*) at phase $p_0$ of its movement. For each ossicle, the data analysis was conducted on sub-volumes manually selected and shown here as yellow rectangles. The transformation extracted for each sub-volume $SV_i$ was expressed with the center of the sub-volume $O^i$ as the origin. **b** A change of basis was needed to express all transformations from the same origin $O^0$ and calculate the mean transformation. The change of basis is explained here taken $SV_0$ as the reference sub-volume. **c** Distribution of the rotation angles and the translation magnitudes extracted for the 7 sub-volumes of the malleus of a fresh-frozen human specimen (*B-fresh3*), stimulated at 120 dB and 128 Hz. The boxplot covers the interquartile interval, crossed by a horizontal line for the median value, and the whiskers show the minimum and maximum values.

Supplementary Figure 3: **Dynamic analyses performed on the temporal bone in a fresh-frozen human specimen (*Fresh3*) stimulated at 128 Hz and 120 dB SPL.**

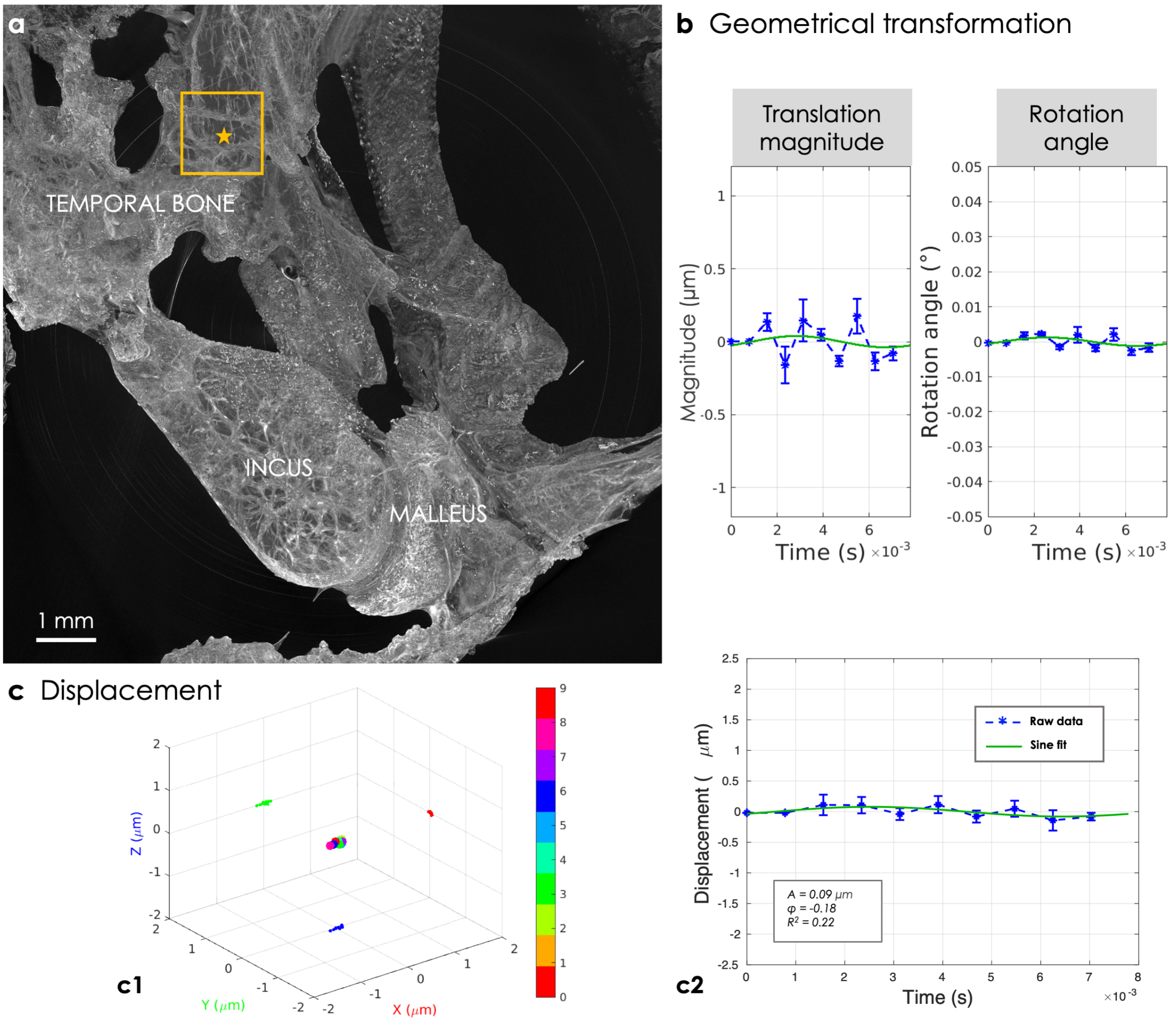


**a** The 3D volume of the middle ear reconstructed at phase $p_0$ is projected in 2D using a maximum intensity projection. **b** Extraction of the mean transformation of four sub-volumes taken from the temporal bone (one sub-volume is shown within the yellow square). **c** The mean displacement of points of interest selected within the temporal bone sub-volumes (yellow star) is shown: **c1** in 3D - each color of the 3D rainbow plot corresponds to a phase of movement (from phase $p_0$ - used as the origin (0,0,0) - to phase $p_9$), and where the 2D projections of the displacements are shown on the XY (blue), YZ (red), and ZX (green) planes. **c2** in 2D - the magnitudes of the displacement vectors are shown over the phases of movement (i.e. over time). The error bars in blue represent the standard deviation of the distribution across the n=4 independent sub-volumes. The maximum displacement magnitude found with our pipeline for a static feature is around 0.3 µm. It gives us the corresponding noise limit of our analyses.
