# Supplementary material for: The human middle ear in motion: 3D visualization and quantification using dynamic synchrotron-based X-ray imaging
Source: Commun Biol. 2024 Feb 7;7:157. doi: 10.1038/s42003-023-05738-6 (PMC10850498; doi:10.1038/s42003-023-05738-6)
Supplement: Supplementary file 2 — Description of Additional Supplementary Files [file 42003_2023_5738_MOESM2_ESM.docx]

**Description of Additional Supplementary Files**

**File name:** Supplementary Movie 1
**Description:** Title: Dynamic post-gated radiography.
Dynamic radiography of a fresh-frozen human specimen (Fresh1) stimulated at 140 dB and 127 Hz. The projections are post-gated into the 10 phases of movement to enable the visualization of the periodic movement of the features, especially the tympanic membrane. Scale bar 1 mm.

**File name:** Supplementary Movie 2
**Description:** Title: 2D dynamic visualization of the ossicular chain.
The static 3D volumes of a fresh-frozen human specimen (Fresh1, stimulated at 140 dB SPL and 127 Hz) were reconstructed for each phase of movement. The ossicular 2D motions were then visualized after reconstruction by selecting the same 2D sections through the 3D volumes. Here, three sections are taken in a plane where the ossicular movement is assumed to be the largest: along the manubrium of the malleus, the long process of the incus, and the crus of the stapes. Scale bar 1 mm

**File name:** Supplementary Movie 3
**Description:** Title: 2D dynamic visualization of the stapes footplate inside the oval window.
The static 3D volumes of a fresh-frozen human specimen (Fresh1, stimulated at 140 dB SPL and 127 Hz) were reconstructed for each phase of movement. Here, a 2D slice within this 4D volume shows the specific movement of the stapes footplate, and emphasizes that the cochlea visible next to it is static. Scale bar 1 mm.

**File name:** Supplementary Movie 4
**Description:** Title: Direct 3D visualization of the ossicular chain motions at 140 dB SPL.
The static 3D volumes of a fresh-frozen human specimen (Fresh1, stimulated at 140 dB SPL and 127 Hz) were reconstructed and segmented for the different phases of movement. Here, phases p0 and p5 are displayed one after the other to show the position difference between these 2 extreme phases of movement.

**File name:** Supplementary Movie 5
**Description:** Title: 3D visualization of the amplified motions of the ossicular chain at 120 dB SPL. Visualization of the ossicular chain movement of a fresh-frozen human specimen (B-Fresh3) subjected to a 120-dB SPL acoustic stimulation. The three ossicles are shown in 4 different phases of motion, p0, p2, p5, and p8. Phases p0 and p5 (in gray and green) almost overlap, while phases p2 and p8 (in blue and yellow) are the two extreme positions of the movement, as expected from Fig. 2(c) and (d).

**File name:** Supplementary Data 1
**Description:** The 5 tabs of the document provide the data behind the graphs of Figure1, Figure3, Figure6, Figure7a-g and Figure7h, respectively. Tab1 (Figure1) gives the intensity profiles (normalized grey values) along the dotted lines depicted in Figure1. Tab2 (Figure3) gives the translations and rotations extracted for 7 different subvolumes in the malleus of sample B-Fresh3 stimulated at 120 dB and 128 Hz. Tab3 (Figure6) gives the displacement vectors found for n=5 to 11 different points selected in the 6 regions of interest of sample B-Fresh3 stimulated at 120 dB and 128 Hz. Tab4 (Figure7a-g) gives the displacement norm as a function of the phase of motion, as well as the displacement amplitude fitted in one cycle of movement, found for n=5 to 11 different points selected in the regions of interest of sample B-Fresh3 stimulated at 128 Hz and 110 dB (3 regions) and 120 dB (6 regions). Tab5 (Figure7h) gives the mean and standard deviation of the displacement amplitude (averaged over the different points) as well as the mean phase fitted in one cycle of motion, and the r2 parameter showing the quality of the sine fit, found for the 6 regions of interest in the 6 samples stimulated at 128 Hz and 2 sound pressure levels, 110 dB and 120 dB.
