# Supplementary figures and images for: The human middle ear in motion: 3D visualization and quantification using dynamic synchrotron-based X-ray imaging
Source: Commun Biol. 2024 Feb 7;7:157. doi: 10.1038/s42003-023-05738-6 (PMC10850498; doi:10.1038/s42003-023-05738-6)

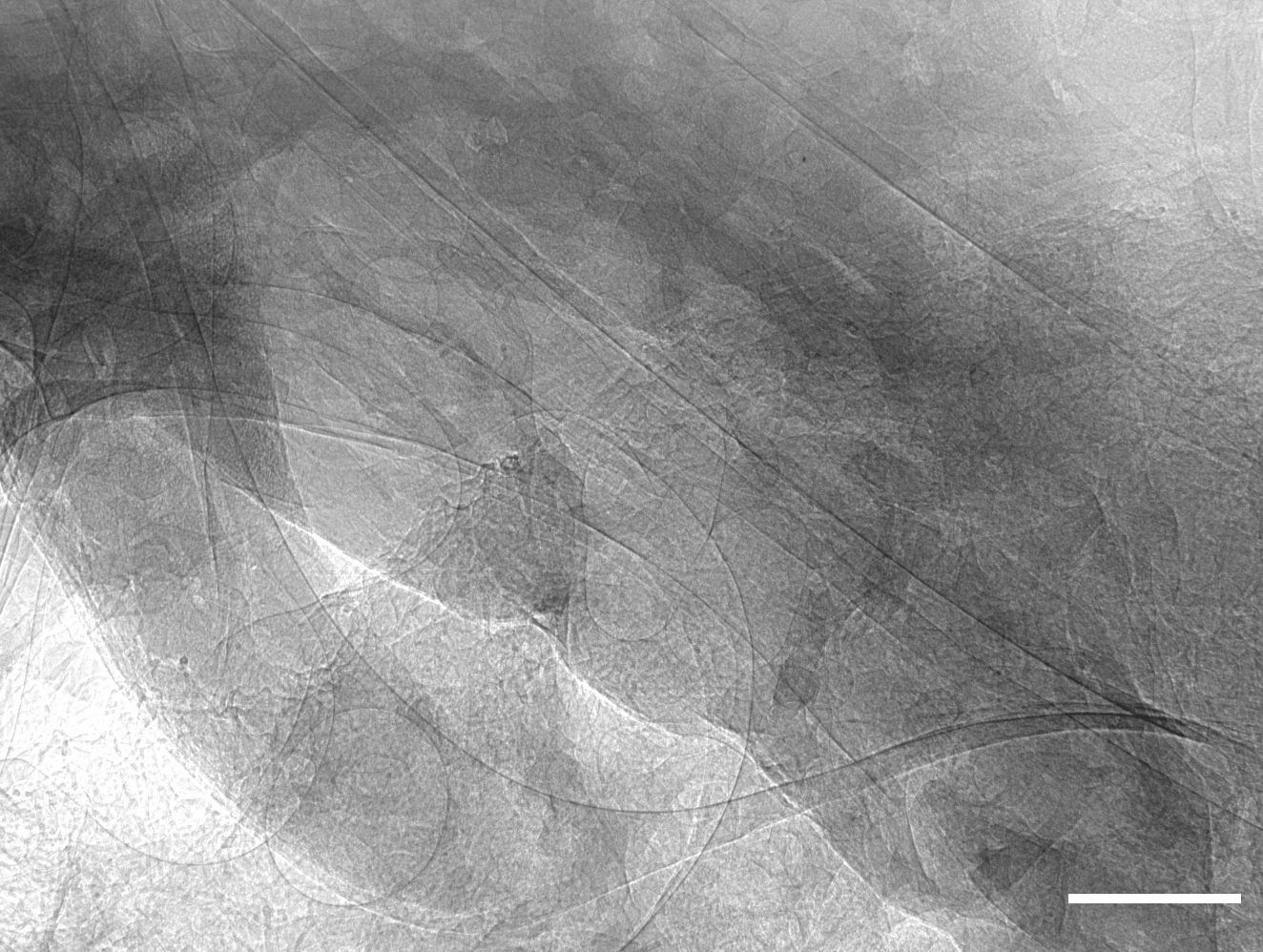

Supplement: Supplementary file 3 — Supplementary Movie 1 [file 42003_2023_5738_MOESM3_ESM.gif]

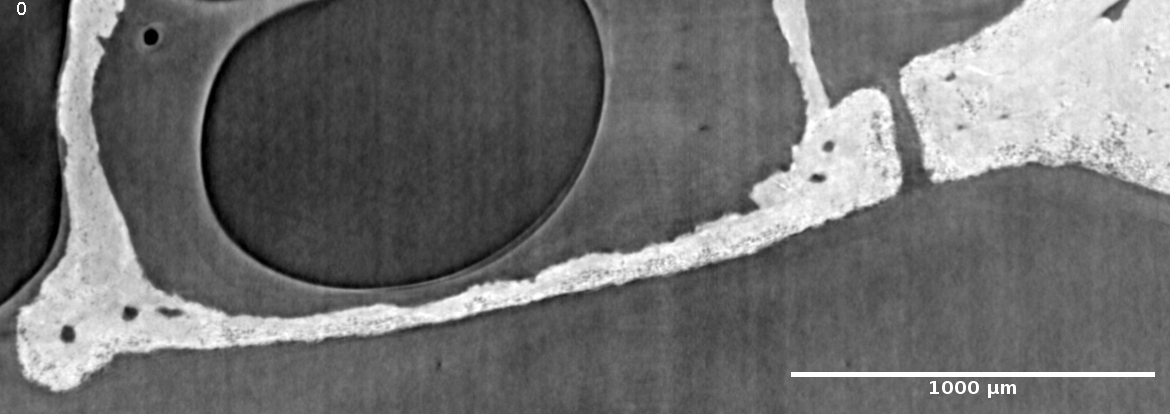

Supplement: Supplementary file 5 — Supplementary Movie 3 [file 42003_2023_5738_MOESM5_ESM.gif]
