# Supplementary material for: The human middle ear in motion: 3D visualization and quantification using dynamic synchrotron-based X-ray imaging
Source: Commun Biol. 2024 Feb 7;7:157. doi: 10.1038/s42003-023-05738-6 (PMC10850498; doi:10.1038/s42003-023-05738-6)
Supplement: Supplementary file 9 — Reporting Summary [file 42003_2023_5738_MOESM9_ESM.pdf]

nature portfolio

Corresponding author(s): Margaux Schmeltz

Last updated by author(s): Dec 15, 2023

Reporting Summary

Nature Portfolio wishes to improve the reproducibility of the work that we publish. This form provides structure for consistency and transparency in reporting. For further information on Nature Portfolio policies, see our Editorial Policies and the Editorial Policy Checklist.

Statistics

For all statistical analyses, confirm that the following items are present in the figure legend, table legend, main text, or Methods section.

n/a	Confirmed	
☐	☒	The exact sample size (*n*) for each experimental group/condition, given as a discrete number and unit of measurement
☐	☒	A statement on whether measurements were taken from distinct samples or whether the same sample was measured repeatedly
☒	☐	The statistical test(s) used AND whether they are one- or two-sided<br>*Only common tests should be described solely by name; describe more complex techniques in the Methods section.*
☒	☐	A description of all covariates tested
☒	☐	A description of any assumptions or corrections, such as tests of normality and adjustment for multiple comparisons
☐	☒	A full description of the statistical parameters including central tendency (e.g. means) or other basic estimates (e.g. regression coefficient) AND variation (e.g. standard deviation) or associated estimates of uncertainty (e.g. confidence intervals)
☒	☐	For null hypothesis testing, the test statistic (e.g. *F*, *t*, *r*) with confidence intervals, effect sizes, degrees of freedom and *P* value noted<br>*Give P values as exact values whenever suitable.*
☒	☐	For Bayesian analysis, information on the choice of priors and Markov chain Monte Carlo settings
☒	☐	For hierarchical and complex designs, identification of the appropriate level for tests and full reporting of outcomes
☒	☐	Estimates of effect sizes (e.g. Cohen's *d*, Pearson's *r*), indicating how they were calculated

Our web collection on statistics for biologists contains articles on many of the points above.

Software and code

Policy information about availability of computer code

Data collection	In this study, we collected the data with custom python codes developed at the TOMCAT beamline (Swiss Light Source, PSI), written by C. Schlepütz, F. Marone and A. Bonnin.
Data analysis	In this study, we analyzed the data with in-house developed python codes to post-gate the tomography projections, written by C. Schlepütz, and an in-house developed matlab pipeline to extract the transformations, written by M. Schmeltz.<br>The Amira-Avizo software (Thermo Scientific Co., version 2020.3.1) with the XImagePAQ – Advanced Image processing and quantification extension was used for 3D and 4D visualization.

For manuscripts utilizing custom algorithms or software that are central to the research but not yet described in published literature, software must be made available to editors and reviewers. We strongly encourage code deposition in a community repository (e.g. GitHub). See the Nature Portfolio guidelines for submitting code & software for further information.

Data

Policy information about availability of data

All manuscripts must include a data availability statement. This statement should provide the following information, where applicable:

- Accession codes, unique identifiers, or web links for publicly available datasets
- A description of any restrictions on data availability
- For clinical datasets or third party data, please ensure that the statement adheres to our policy

The raw data supporting the conclusions of this article have been archived and published in agreement with the FAIR principles, on the SciCat Data Catalog of PSI. They are openly available via a DOI provided in the references, and reproduced here: https://doi.org/10.16907/e4ae0d62-c7a1-4743-8d46-207e431be4ac. The derived measurements underlying the graphs in the manuscript are provided in Supplementary Data 1.

Research involving human participants, their data, or biological material

Policy information about studies with human participants or human data. See also policy information about sex, gender (identity/presentation), and sexual orientation and race, ethnicity and racism.

Reporting on sex and gender	Neither sex nor gender information were collected in this study.
Reporting on race, ethnicity, or other socially relevant groupings	No information about race, ethnicity or other social groupings were collected in this study.
Population characteristics	No population characteristics were collected in this study.
Recruitment	Seven human fresh-frozen temporal bones were obtained from anonymous body donors: four from the institute of pathology in Bern, Switzerland and three from the Eaton Peabody Laboratories, Mass Eye and Ear, Boston, MA, USA.
Ethics oversight	The study protocol was approved by the local ethical committee of Bern (Kantonale Ethikkommission Bern, KEK-BE 2016-00887) and the local ethical committee of the Paul Scherrer Institute (Ethikkommission Nordwest-und Zentralschweiz, 2017-00805), as well as the Mass General Brigham Institutional Review Board (#2022P001306).

Note that full information on the approval of the study protocol must also be provided in the manuscript.

Field-specific reporting

Please select the one below that is the best fit for your research. If you are not sure, read the appropriate sections before making your selection.

☒ Life sciences ☐ Behavioural & social sciences ☐ Ecological, evolutionary & environmental sciences

For a reference copy of the document with all sections, see nature.com/documents/nr-reporting-summary-flat.pdf

Life sciences study design

All studies must disclose on these points even when the disclosure is negative.

Sample size	Seven human fresh-frozen temporal bones were obtained from anonymous body donors. It was the maximal sample size available as human temporal bones coming from body donor are quite rare. The sample size is enough to draw tendencies while having an idea of the inter-sample dispersion.
Data exclusions	Two additional human fresh-frozen temporal bones were measured but excluded from this study as the sound calibration performed to measure the sound pressure level applied in the ear canal was wrong (probe microphone damaged).
Replication	The reproducibility of our findings has been assessed by repeating the same experiment on the seven samples available.
Randomization	No experimental groups were used in this study.
Blinding	No experimental groups were used in this study.

Reporting for specific materials, systems and methods

We require information from authors about some types of materials, experimental systems and methods used in many studies. Here, indicate whether each material, system or method listed is relevant to your study. If you are not sure if a list item applies to your research, read the appropriate section before selecting a response.

Materials & experimental systems

n/a	Involved in the study
☒	☐ Antibodies
☒	☐ Eukaryotic cell lines
☒	☐ Palaeontology and archaeology
☒	☐ Animals and other organisms
☒	☐ Clinical data
☒	☐ Dual use research of concern
☒	☐ Plants

Methods

n/a	Involved in the study
☒	☐ ChIP-seq
☒	☐ Flow cytometry
☒	☐ MRI-based neuroimaging
